# Discrimination‐Based Continuous Traumatic Stress, PTSD and Chronic Pain in Syrian Refugees: A Moderated Mediation Analysis

**DOI:** 10.1002/cpp.70133

**Published:** 2025-07-23

**Authors:** Emre Han Alpay, Ibrahim Aref Kira

**Affiliations:** ^1^ Department of Psychology Mersin University Mersin Türkiye; ^2^ Center for Stress, Trauma, and Resilience Georgia State University Atlanta Georgia USA

**Keywords:** chronic pain, continuous traumatic stress, discrimination, PTSD, somatic symptoms

## Abstract

**Purpose:**

This study aimed to examine the relationships among continuous traumatic stressors (CTS), posttraumatic stress disorder (PTSD), somatic symptoms, and chronic pain in a sample of Syrian refugees. Specifically, we hypothesized that PTSD would mediate the association between discrimination‐based CTS and chronic pain and that somatic symptoms would moderate the link between PTSD and chronic pain—such that higher somatic symptom levels would amplify this association.

**Materials and Methods:**

A total of 669 Syrian refugees residing in Türkiye participated in the study. Data were collected through face‐to‐face interviews using validated self‐report instruments, including the Patient Health Questionnaire‐15 (PHQ‐15), the PTSD Checklist for DSM‐5 (PCL‐5), the Short‐Form McGill Pain Questionnaire (SF‐MPQ) and the Cumulative Trauma Scale.

**Results:**

PTSD symptoms mediated the relationship between discrimination‐based CTS and chronic pain. Additionally, somatic symptoms significantly moderated the association between PTSD and chronic pain, such that individuals with higher levels of somatic symptoms exhibited a stronger link between PTSD symptoms and chronic pain severity.

**Conclusion:**

These findings emphasize the relationship between discrimination‐based CTS, psychological distress, and physical complaints among Syrian refugees in Türkiye. While the results provide important insights into trauma‐related health outcomes in this group, caution should be exercised in generalizing the findings to all displaced populations. The results highlight the profound impact of prolonged traumatic stress on both psychological and physical health and emphasize the need for trauma‐informed, culturally sensitive clinical interventions for displaced individuals.

## Summary

- Discrimination‐based continuous traumatic stress (CTS) is a critical yet under‐recognized contributor to PTSD and chronic pain among refugees. Practitioners should assess for chronic identity‐based stressors—including ongoing discrimination—as part of a trauma‐informed clinical evaluation.
- PTSD symptoms serve as a central mechanism linking CTS to chronic pain. Clinicians must be aware that unaddressed PTSD can manifest in physical symptoms, particularly chronic pain.
- Somatic symptoms significantly amplify the impact of PTSD on chronic pain. In populations where psychological distress is expressed somatically, elevated somatic symptomatology may indicate more severe underlying trauma and pain dysregulation. Practitioners should interpret somatic complaints not merely as physical issues but as potential markers of unresolved trauma.
- Cultural and contextual sensitivity is essential in clinical encounters. In refugee populations, stigma surrounding mental health may lead individuals to express emotional suffering through bodily symptoms. Clinicians should adopt culturally attuned communication strategies to explore these complaints empathetically and without pathologizing.
- Integrated care approaches are necessary. Collaborative models involving mental health professionals, primary care providers and community organizations can help address the intertwined psychological, somatic and social needs of forcibly displaced individuals.

The armed conflict that began in Syria in 2011 triggered one of the largest refugee crises in recent history, forcing millions of Syrians to seek refuge in neighbouring countries, including Türkiye, Lebanon, Iraq, Egypt and Jordan. These host countries continue to face significant humanitarian challenges as a result of the crisis. By December 2022, an estimated 13 million Syrians had been displaced, and at least 580,000 lives had been lost (United Nations High Commissioner for Human Rights 2023). Refugees are frequently exposed to multiple traumatic events before, during, and after displacement (Cantekin and Gençöz 2017; Rawers et al. 2024; Teodorescu et al. 2012). The cumulative impact of these stressors contributes to elevated rates of mental health disorders, including post‐traumatic stress disorder (PTSD), depression, anxiety disorders, somatic symptoms and chronic pain (Alpay et al. 2021; Blackmore et al. 2020; Fazel et al. 2005; Kira et al. 2023).

Trauma research has traditionally categorized traumatic experiences into two primary types: Type I trauma, which refers to single, acute traumatic events, and Type II trauma, which encompasses multiple or repeated traumas over time (Terr 1991; Herman 1992). Type I trauma typically involves discrete incidents such as accidents or natural disasters, whereas Type II trauma is often associated with chronic exposure to interpersonal violence or abuse, leading to more complex psychological outcomes. While these frameworks have advanced our understanding of trauma, they often fall short in capturing the chronic, identity‐based and structurally embedded forms of adversity experienced by marginalized populations. To address this gap, the continuous traumatic stress (CTS) model (Kira et al. 2013; Kira 2021) was developed to conceptualize chronic, evolving exposure to trauma without a predictable endpoint. CTS is characterized by cumulative and persistent stressors, wherein individuals are repeatedly subjected to life‐threatening situations, oppression, displacement and socio‐economic hardship (Kira et al. 2015; Kira, Fawzi, et al. 2019). Unlike acute trauma, CTS does not allow for periods of recovery, resulting in prolonged activation of stress‐response systems and increased vulnerability to mental health disorders, somatic symptoms and chronic pain syndromes (Kira, Shuwiekh, et al. 2019). This framework has proven particularly useful for understanding the experiences of minority groups such as refugees and displaced populations. These populations are exposed to traumatic events before migration (e.g., war and persecution), during migration (e.g., displacement and loss of identity) and after migration (e.g., discrimination, legal insecurity and economic hardship) (Andisha and Lueger‐Schuster 2024; Feyissa et al. 2022; Goodkind et al. 2021; Theisen‐Womersley 2021).

## 1 The Psychological and Physiological Impact of Continuous Traumatic Stress: A Focus on Discrimination

Discrimination can be defined as the unfair or prejudiced treatment of individuals because of their membership of a stigmatized social group. It may stem from ascribed identities, such as race, ethnicity, gender age or achieved identities, including social class, educational attainment or occupational status (Williams et al. 2018). Discrimination systematically disadvantages particular social groups, severely constraining their potential for achievement, creativity and productivity (Williams and Mohammed 2009). The effects of discrimination are exacerbated when multiple forms of oppression intersect, such as those based on gender, race, class and sexuality, producing a compounded and pervasive form of marginalization (Crenshaw 2013). Given its chronic, unpredictable and inescapable nature, discrimination has increasingly been conceptualized as a form of CTS (Kira et al. 2013; Kira 2021), particularly in contexts involving long‐term marginalization.

Empirical evidence suggests that chronic exposure to discrimination is linked to elevated rates of PTSD, complex PTSD (CPTSD), depression, and anxiety (Campo‐Arias et al. 2022; Kira et al. 2023; McClendon et al. 2021). Prolonged social exclusion, racial trauma or gender‐based oppression compromises neurobiological and psychological regulation (McEwen 2004; Sapolsky 2015), particularly through hyperactivation of the hypothalamic–pituitary–adrenal (HPA) axis and dysregulation of the autonomic nervous system (ANS). Individuals who internalize or continuously confront discriminatory environments often display elevated inflammatory markers, aberrant cortisol rhythms and impaired immune functioning, which have been associated with increased pain sensitivity, autoimmune disorders, and cardiovascular dysfunction (Cuevas et al. 2020; Miller et al. 2007; Saban et al. 2018). A frequently overlooked consequence of discrimination‐related CTS is its association with chronic pain syndromes, particularly among refugees and racialized minority groups who experience layered adversities throughout the migration trajectory. Research has shown that individuals exposed to such stressors report elevated rates of medically unexplained pain—including fibromyalgia, chronic headaches and musculoskeletal pain (Altun et al. 2022; Vermeir et al. 2021). This association is mediated through both physiological mechanisms, such as stress‐induced neuroinflammation and altered pain processing (Gilliam et al. 2019), as well as psychological pathways, including PTSD (McKernan et al. 2019). Critically, PTSD symptoms often mediate the relationship between exposure to traumatic events and chronic pain (Häuser et al. 2019; McKernan et al. 2019; Karimov‐Zwienenberg et al. 2024). Trauma‐induced hypervigilance, emotional dysregulation and autonomic rigidity are believed to lower pain thresholds and intensify bodily distress (Tesarz et al. 2020). These findings converge to suggest that CTS constitutes a biopsychosocial trauma that not only disrupts mental health but also alters pain perception and bodily integrity—even in the absence of physical injury (Van Houdenhove and Luyten 2005).

## 2 The Moderating Role of Somatic Symptoms

While PTSD may mediate the trauma–pain relationship, somatic symptoms—the tendency to express psychological distress through physical complaints—may further exacerbate this connection (Henningsen et al. 2018). Somatic symptoms are particularly prevalent among trauma‐exposed populations, where cultural and psychological factors influence how distress is communicated. In many collectivistic or trauma‐affected societies, emotional suffering is often expressed somatically due to cultural norms that discourage open discussion of psychological issues or emotional vulnerability (Hinton and Lewis‐Fernández 2011; Kirmayer and Sartorius 2007). Furthermore, mental health stigma may prevent individuals from acknowledging or seeking help for emotional symptoms, leading to the presentation of distress in the form of physical ailments such as chronic pain, fatigue or gastrointestinal problems. This somatic mode of symptom expression may serve as a culturally acceptable pathway for seeking help or legitimizing distress. Research has shown that individuals with high levels of somatic symptoms are more likely to experience severe and widespread pain, suggesting that somatic symptoms may amplify the PTSD–chronic pain link by reinforcing maladaptive pain perceptions and pain‐related distress (Morasco et al. 2013; Villano et al. 2007). A study by Kira et al. (2013) found that refugees with high somatic symptom scores reported significantly more severe pain symptoms, even after controlling for PTSD severity. This suggests that somatic symptoms not only coexist with PTSD but also interact with it, leading to a feedback loop where psychological distress manifests as physical symptoms, which in turn reinforce emotional suffering (Henningsen et al. 2018).

Despite the growing recognition of discrimination as a form of continuous traumatic stress, few studies have empirically investigated its biopsychosocial consequences in forcibly displaced populations using integrative analytical models. In particular, the mechanisms through which discrimination‐based CTS leads to chronic physical symptoms remain unclear. This study addresses this knowledge gap by examining post‐traumatic stress disorder (PTSD) as a psychological mediator and somatic symptoms as cultural and physiological moderators in the relationship between discrimination‐based trauma and chronic pain. The refugee context provides a critical lens through which to understand the intersection of sociopolitical exclusion, trauma exposure, and embodied distress. By focusing on Syrian refugees, a population disproportionately affected by multiple stressors, this study aims to improve our understanding of trauma‐related physical health outcomes in marginalized communities.

## 3 Aim of the Study

The aim of this study is to investigate the relationship between continuous traumatic stressors, particularly discrimination, PTSD symptoms, somatic symptoms and chronic pain among Syrian refugees. Specifically, the study has three primary objectives:

First, it aims to examine the direct effects of continuous traumatic stressors, especially those rooted in discrimination, on PTSD symptoms and chronic pain. Given that CTS involves ongoing and cumulative stressors without a foreseeable end—such as systemic discrimination, marginalization and social exclusion—it is critical to evaluate their impact on both psychological and physiological health outcomes in forcibly displaced populations.

Second, the study seeks to determine whether PTSD symptoms mediate the relationship between continuous traumatic stressors and chronic pain. Since PTSD frequently emerges as a consequence of prolonged trauma exposure, understanding its mediating role may offer insights into how psychological distress serves as a pathway to chronic somatic suffering.

Finally, the study explores the moderating role of somatic symptoms in the relationship between PTSD symptoms and chronic pain. Somatic symptoms—such as fatigue, headaches and gastrointestinal distress—are highly prevalent among trauma‐exposed individuals and may amplify the subjective experience of pain. Investigating this moderating effect will help clarify whether individuals with higher levels of somatic symptoms experience a stronger association between PTSD and chronic pain.

**FIGURE 1:**
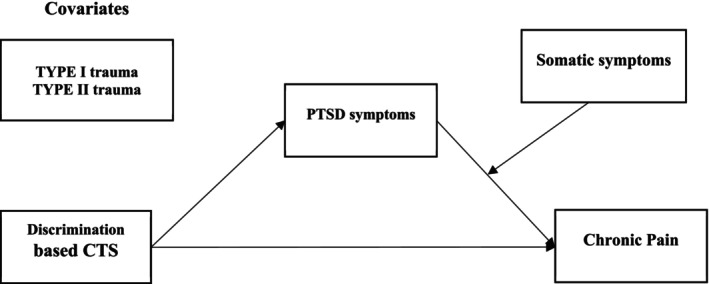
Proposed model.

The proposed moderated mediation model is illustrated in Figure 1.

Based on these objectives, the following hypotheses will be tested:

Hypothesis 1 *Discrimination‐based CTS are positively associated with PTSD symptoms in Syrian refugees*.

Hypothesis 2 *PTSD symptoms are positively associated with chronic pain severity*.

Hypothesis 3 *Discrimination‐based CTS has an indirect effect on chronic pain through PTSD symptoms*.

Hypothesis 4 *Somatic symptoms moderate the relationship between PTSD symptoms and chronic pain*.

## 4 Methods

### 4.1 Participants and Procedure

The study sample consisted of 669 adult Syrian refugees residing in Mersin, Türkiye, aged between 18 and 69 years (*M* = 32.34, SD = 10.85). Of these, 434 (65.4%) identified as women and 230 (34.6%) as men. Regarding marital status, 49.1% (*n* = 326) were married, 38.3% (*n* = 254) were single, 2% (*n* = 13) were divorced and 10.7% (*n* = 71) were widowed.

Data collection took place between 13 May and 8 September 2023 through face‐to‐face interviews conducted in refugee‐populated areas of Mersin. To facilitate the process, five bilingual Syrian interviewers—employed by non‐governmental organizations (NGOs) as translators—were recruited. All interviewers were fluent in both Arabic and Turkish, ensuring effective communication with participants. Prior to data collection, one of the study's authors provided a 1‐day training session to the interviewers, covering the study's objectives, ethical protocols, and data collection procedures. During the interviews, participants were first informed about the study's purpose and ethical guidelines. They were then given a self‐administered questionnaire in Arabic. For participants with low literacy levels, interviewers provided assistance in reading and completing the questionnaire.

The study was conducted in accordance with strict ethical standards and received approval from the Mersin University Social Sciences Ethics Committee. Informed consent was obtained from all participants prior to participation. The inclusion criteria were as follows: (a) exposure to at least one traumatic event (as defined by the Cumulative Trauma Scale), (b) having fled to Türkiye after the onset of the Syrian civil war and (c) being at least 18 years of age. All participants reported exposure to at least one traumatic event that met Criterion A of the DSM‐5. As a result, no participants were excluded based on trauma exposure, and the full sample was included in the analyses.

### 4.2 Measures

#### 4.2.1 Patient Health Questionnaire‐15 (PHQ‐15)

The PHQ‐15 is a 15‐item self‐report scale designed to assess somatic symptoms experienced over the past 4 weeks (Kroenke et al. 2002). Each item is rated on a 3‐point Likert scale (0 = *not bothered at all* to 2 = *bothered a lot*). Previous studies (e.g., Zijlema et al. 2013) have demonstrated that the PHQ‐15 is a reliable and valid measure for evaluating somatic symptoms. The Arabic translation of the PHQ‐15, validated by Alhadi et al. (2017), reported a Cronbach's alpha internal consistency of 0.83. In the present study, the scale demonstrated good internal consistency, with a Cronbach's alpha of 0.88.

#### 4.2.2 Posttraumatic Stress Disorder Checklist‐5 (PCL‐5)

The PCL‐5 is a 20‐item self‐report measure that assesses PTSD symptom severity over the past month (Weathers et al. 2013). It aligns with the DSM‐5 symptom clusters: avoidance, intrusions, negative alterations in cognitions and mood and alterations in arousal and reactivity. Participants rated the severity of their symptoms over the past month using a 5‐point scale, with responses ranging from 0 (*not at all*) to 4 (*extremely*). Ibrahim et al. (2018) translated the PCL‐5 into Arabic, reporting strong internal consistency. In the current study, we calculated Cronbach's alpha for the total PCL‐5 score to be 0.96, reflecting good reliability.

#### 4.2.3 Short‐Form McGill Pain Questionnaire (SF‐MPQ)

Developed by Melzack (1987), the SF‐MPQ is a widely used self‐report instrument for assessing chronic pain experience. It comprises 15 items divided into two dimensions: sensory (11 items) and affective (4 items). Participants rate pain intensity on a 4‐point Likert scale (0 = *none*, 3 = *severe*). Terkawi et al. (2017) translated the SF‐MPQ into Arabic and reported good internal consistency (α = 0.85). In the present study, Cronbach's alpha internal consistency score for the total score is 0.95.

#### 4.2.4 Cumulative Trauma Scale (CST‐S)

The *Cumulative Stressors and Traumas Scale—Short Form* (CST‐S; Kira 2001, 2021; Kira et al. 2008) was developed to assess exposure to a broad range of traumatic stressors within the Development‐Based Trauma Framework (DBTF). It includes seven categories of trauma: collective identity trauma (e.g., discrimination), personal identity trauma (e.g., childhood abuse), role identity or achievement trauma (e.g., academic or occupational failure), survival trauma (e.g., disasters, accidents), attachment trauma (e.g., parental abandonment), secondary trauma (indirect exposure through close others) and gender discrimination. In the present study, we utilized the intersected discrimination subscale, which consists of five items assessing experiences of discrimination based on gender (e.g., being put down, denied rights or treated unfairly by society or institutions due to one's gender), sexual orientation (e.g., discrimination‐based on sexual preference), social group membership (e.g., being targeted due to ethnicity, race, culture, religion or national origin) and genocidal targeting (e.g., having a racial background with a history of oppression or genocide). Participants responded to each item using a 5‐point Likert scale ranging from 0 (*never*) to 4 (*many times*). In this study, the internal consistency of the cumulative trauma occurrence subscale was excellent, with a Cronbach's alpha of 0.89.

### 4.3 Statistical Analysis

We first examined missing values. Given that the proportion of missing data was low (< 5%) and determined to be missing completely at random (MCAR), we employed mean imputation to replace missing values using the variable's mean based on available scores. This method was selected due to its simplicity and minimal bias under MCAR conditions. To detect multivariate outliers, we calculated Mahalanobis distances. Normality assumptions were evaluated by examining skewness and kurtosis scores for all variables. As these values fell within the acceptable range of ±2 (George and Mallery 2018), the variables were considered normally distributed. Descriptive statistics, including means, standard deviations and frequencies, were calculated to summarize the characteristics of the sample. Pearson correlation analyses were then conducted to examine bivariate relationships among variables. To test the hypothesized moderated mediation model, we performed a two‐step regression analysis using the PROCESS macro in SPSS Statistics version 26 (Hayes 2013). First, a mediation analysis was conducted to assess whether PTSD symptoms mediated the relationship between discrimination‐based CTS and chronic pain. Indirect effects were tested using bootstrapping procedures with 5000 samples, and 95% bias‐corrected bootstrapped confidence intervals (CIs) were used to determine statistical significance (Preacher and Hayes 2008). Second, a moderated mediation analysis was conducted to examine whether somatic symptoms moderated the association between PTSD symptoms and chronic pain. An interaction term (PTSD × somatic symptoms) was created and included in the model. Conditional effects were examined at low (−1 SD), mean and high (+1 SD) levels of somatic symptoms. To enhance interpretability, significant interaction effects were plotted to visualize the conditional relationship between PTSD symptoms and chronic pain across varying levels of somatic symptoms. Moreover, we added Type I and Type II traumas as covariates in all analyses to statistically control for their potential confounding influence on the relationships among the primary study variables.

### 4.4 Preliminary Analyses

First, the means, standard deviations and minimum and maximum values of the study variables were examined. These descriptive statistics are presented in Table 1.

**TABLE 1:** Descriptive statistics of the study variables.

	Min	Max	Mean	Sd
Somatic symptoms	00	30.00	7.30	6.95
PTSD symptoms	00	73.00	20.97	19.47
Chronic pain	00	45.00	7.09	9.86
Discrimination‐based CTS	00	6.00	2.35	1.44
Type I trauma	00	4.00	1.24	0.56
Type II trauma	00	6.00	1.23	1.06

*Note: N* = 669.

Abbreviation: PTSD: post‐traumatic stress disorder.

We conducted a correlation analysis among variables before testing our hypotheses. Table 2 displays Pearson correlation coefficients and the descriptive statistics of the variables. All variables were positively correlated with each other.

**TABLE 2:** Correlation coefficients among variables.

	1	2	3	4	5	6
1. PTSD	—	0.596**	0.609**	0.526**	0.532**	0.283**
2. Somatic symptoms	—	—	0.715**	0.420**	0.398**	0.183**
3. Chronic pain	—	—	—	365**	0.373**	0.220**
4. Discrimination	—	—	—	—	0.462**	0.401**
5. Type I trauma	—	—	—	—	—	0.355**
6. Type II trauma	—	—	—	—	—	—

*Note: N* = 669.

Abbreviation: PTSD: post‐traumatic stress disorder.

** *p* < 0.001.

### 4.5 PTSD as a Mediator in the Link Between Discrimination‐Based CTS and Chronic Pain

A mediation analysis was conducted using Model 4 of the PROCESS macro (Hayes 2013) to examine whether PTSD symptoms mediate the relationship between discrimination‐based continuous traumatic stress (CTS) and chronic pain in a sample of 669 adult Syrian refugee participants. Type I and Type II trauma exposures were included in the model as covariates. Results showed that the model accounted for 37.63% of the variance in chronic pain (*R* ^2^ = 0.3763). Discrimination‐based CTS significantly predicted PTSD symptoms (*b* = 4.68, SE = 0.48, *t* = 9.86, *p* < 0.001, 95% CI [3.75, 5.62]). In turn, PTSD symptoms were significantly associated with chronic pain (*b* = 0.28, SE = 0.02, *t* = 14.18, *p* < 0.001, 95% CI [0.24, 0.32]), controlling for discrimination‐based CTS and covariates. The direct effect of discrimination‐based CTS on chronic pain was non‐significant (*b* = 0.26, SE = 0.26, *t* = 1.01, *p* = 0.313, 95% CI [−0.25, 0.77]), whereas the indirect effect of discrimination‐based CTS on chronic pain via PTSD symptoms was significant (*b* = 1.31, Boot SE = 0.19, 95% Boot CI [0.96, 1.71]). Lastly, neither Type I nor Type II trauma emerged as significant covariates in the model (Type I trauma: *b* = 0.34, *p* = 0.196; Type II trauma: *b* = 0.55, *p* = 0.376).

### 4.6 Discrimination‐Based Continuous Traumatic Stressors, PTSD, Somatic Symptoms and Chronic Pain: Testing a Moderated Mediation Model

A moderated mediation analysis was conducted using Model 14 of the PROCESS macro to examine whether the indirect effect of discrimination‐based CTS on chronic pain through PTSD symptoms varies depending on levels of somatic symptoms. The analysis included 669 adult Syrian refugee participants, and Type I and Type II trauma exposures were entered as covariates. The overall model was statistically significant and accounted for 59.12% of the variance in chronic pain, *R* ^2^ = 0.5912, *F*(6, 662) = 159.58, *p* < 0.001.

Discrimination‐based CTS significantly predicted PTSD symptoms (*b* = 4.68, SE = 0.48, *t* = 9.86, *p* < 0.001, 95% CI [3.75, 5.62]). In turn, PTSD symptoms were significantly associated with chronic pain (*b* = 0.13, SE = 0.02, *t* = 7.35, *p* < 0.001, 95% CI [0.10, 0.17]). Somatic symptoms also emerged as a robust predictor of chronic pain (*b* = 0.71, SE = 0.05, *t* = 15.33, *p* < 0.001, 95% CI [0.62, 0.80]). Furthermore, the interaction between PTSD symptoms and somatic symptoms was significant (*b* = 0.012, SE = 0.002, *t* = 6.46, *p* < 0.001, 95% CI [0.0082, 0.0154]), indicating that the strength of the association between PTSD symptoms and chronic pain was greater among individuals with higher levels of somatic symptoms.

**FIGURE 2:**
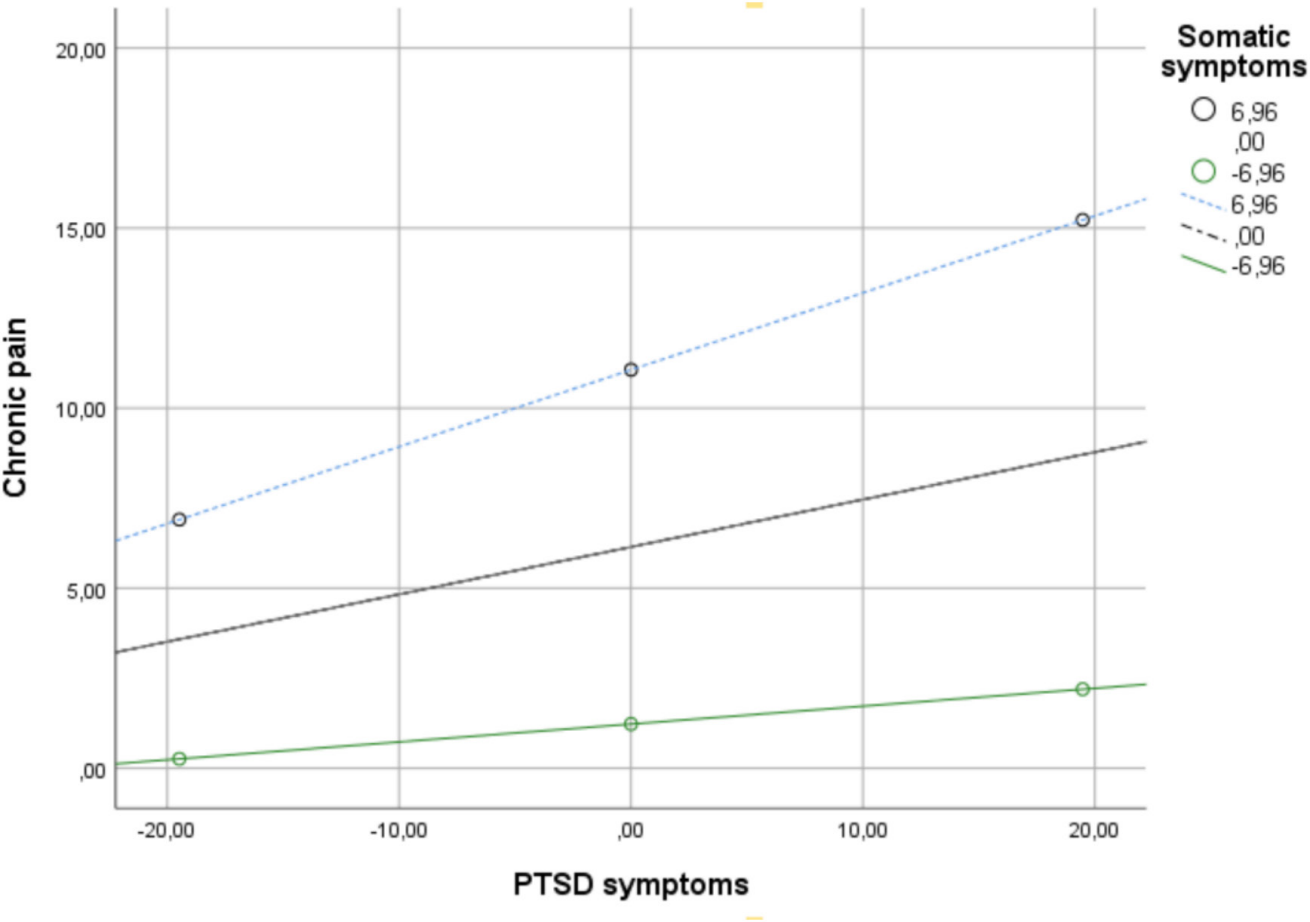
Moderating effect of somatic symptoms on the PTSD–chronic pain association.

The direct effect of discrimination‐based CTS on chronic pain was not statistically significant after accounting for the mediating role of PTSD symptoms (*b* = −0.36, SE = 0.21, *t* = −1.67, *p* = 0.096, 95% CI [−0.77, 0.06]). However, 5000 resample bootstrap analysis results showed that conditional indirect effect analyses revealed that PTSD symptoms significantly mediated the relationship between discrimination‐based CTS and chronic pain, and this indirect effect was moderated by somatic symptom levels. As illustrated in Figure 2, at low levels of somatic symptoms (−1 SD), the indirect effect was significant but relatively weak (*b* = 0.23, Boot SE = 0.11, 95% Boot CI [0.035, 0.47]). At mean levels of somatic symptoms, the indirect effect increased in strength (*b* = 0.62, Boot SE = 0.11, 95% Boot CI [0.42, 0.86]). At high levels of somatic symptoms (+1 SD), the indirect effect was strongest (*b* = 1.00, Boot SE = 0.17, 95% Boot CI [0.70, 1.35]).

The index of moderated mediation was statistically significant (index = 0.055, SE = 0.012, 95% CI [0.033, 0.080]), indicating that the indirect effect of discrimination‐based CTS on chronic pain through PTSD significantly varied depending on levels of somatic symptoms. These findings support a moderated mediation model in which PTSD symptoms mediate the effect of discrimination‐related trauma on pain, and somatic symptoms exacerbate this indirect pathway.

Regarding covariates, neither Type I trauma nor Type II trauma emerged as significant predictors of chronic pain in the moderated mediation model. Specifically, Type I trauma was not significantly associated with pain (*b* = 0.15, SE = 0.21, *t* = 0.71, *p* = 0.475), nor was Type II trauma (*b* = 0.73, SE = 0.51, *t* = 1.43, *p* = 0.153). These results suggest that, in this model, the unique effects of acute and developmental trauma types on chronic pain were not statistically meaningful after accounting for the effect of PTSD symptoms, discrimination‐based CTS and somatic symptoms.

## 5 Discussion

The present study investigated the relationship between discrimination‐based CTS, PTSD symptoms, somatic symptoms and chronic pain in a sample of Syrian refugees living in Türkiye. Drawing on the framework of continuous traumatic stress, we proposed and tested a moderated mediation model in which PTSD symptoms mediated the relationship between discrimination‐based CTS and chronic pain, and somatic symptoms moderated the association between PTSD and pain. We believe that our findings provide insights into the interconnected nature of exposure to discrimination, psychological distress and somatic outcomes within refugee populations.

Consistent with our first hypothesis (Hypothesis 1), exposure to discrimination‐based CTS was significantly associated with increased PTSD symptom severity. This finding aligns with prior research demonstrating that chronic, unpredictable and uncontrollable trauma—particularly when rooted in identity‐based discrimination such as racism, sexism or refugee status—contributes to heightened PTSD symptoms (Ahmad et al. 2024; Dadras and Diaz 2024; Hruschak and Cochran 2018). The CTS framework, particularly in the context of structural and interpersonal discrimination, involves sustained threat and lacks temporal closure, which may hinder the psychological processing and resolution of traumatic experiences (Kira 2021). Unlike acute traumas, discrimination‐based CTS is cumulative and often anticipatory, leading to prolonged hypervigilance and emotional dysregulation (Carter 2007). While such chronic exposures may not directly cause PTSD, they may intensify pre‐existing symptoms, inhibit recovery or reactivate dormant trauma responses, as evidenced by prior studies focusing on refugees and marginalized populations (Nickerson et al. 2015; Silove et al. 2017).

Supporting our second and third hypotheses (Hypotheses 2 and 3), PTSD symptoms were positively associated with chronic pain severity and mediated the relationship between CTS and chronic pain. These findings are consistent with a growing body of literature suggesting that PTSD contributes to altered pain processing and increased vulnerability to somatic syndromes (McKernan et al. 2019; Häuser et al. 2019). Neurobiological research indicates that trauma‐induced dysregulation of the HPA axis and sensitization of central pain pathways may underlie the co‐occurrence of PTSD and chronic pain (Miller et al. 2007; Gądek‐Michalska et al. 2013). Furthermore, PTSD‐related features such as hyperarousal, emotional numbing and attentional biases towards threat may amplify pain perception and reduce pain tolerance (Gómez‐Pérez et al. 2015; Strigo et al. 2010).

Interestingly, there was no statistically significant direct association between discrimination‐based CTS and chronic pain, suggesting that the impact of discrimination‐based CTS on physical symptoms may be largely indirect, operating through trauma‐related psychological mechanisms such as PTSD. This finding reinforces the idea that PTSD is a key mediator in the embodiment of psychosocial stressors—a process whereby chronic emotional suffering manifests in the body through neurobiological dysregulation. The results are also consistent with previous research indicating that discrimination alone may not cause somatic outcomes unless accompanied by significant psychological distress (Dadras and Diaz 2024). Specifically, chronic discrimination may sensitize individuals to threat by activating the HPA axis and limbic circuits over a prolonged period. When coupled with unprocessed trauma, this can facilitate central sensitization and pain amplification (McEwen 2004; Gądek‐Michalska et al. 2013). This highlights the importance of assessing PTSD symptoms as both clinical outcomes and psychophysiological pathways linking social adversity to bodily pain.

Our moderated mediation analysis revealed that somatic symptoms significantly strengthened the association between PTSD symptoms and chronic pain, thereby confirming our final hypothesis (Hypothesis 4). Specifically, the indirect effect of discrimination‐based CTS on chronic pain through PTSD was more pronounced at higher levels of somatic symptoms. This finding echoes previous work suggesting that somatic symptoms act as an amplifier of psychological distress by converting affective arousal into physical complaints (Henningsen et al. 2018; Morasco et al. 2013). In trauma‐exposed populations, particularly within cultural contexts where direct emotional expression is shaped by social norms or constrained by stigma, somatic symptoms may emerge as a socially and culturally mediated mode of expressing distress (Hinton and Lewis‐Fernández 2011; Kirmayer and Sartorius 2007). It is important to recognize that such expressions are not universal but vary significantly across sociocultural settings, shaped by diverse beliefs about the body, suffering and emotional communication. Our results further indicate that individuals with both high PTSD and somatic symptom severity are at elevated risk for experiencing severe and functionally impairing chronic pain.

## 6 Limitations and Future Directions

Despite its contributions, this study has several limitations. First, the cross‐sectional design prevents the drawing of causal inferences between discrimination‐based CTS, PTSD, somatic symptoms and chronic pain. While the statistical models suggest directional relationships, longitudinal studies are essential to confirm temporal ordering and better understand the trajectories of CTS‐related psychological and somatic symptoms over time. These models are not intended to provide evidence of causality, but rather to identify patterns of association that warrant further investigation in longitudinal studies. Second, the sample was limited to Syrian refugees residing in Türkiye, which may restrict the generalisability of the results to other forcibly displaced populations. The cultural, legal and socioeconomic conditions in countries hosting refugees vary widely, and these differences can significantly influence the expression and impact of trauma‐related symptoms. Conducting this study in diverse cultural and geopolitical contexts would enhance the external validity and cross‐cultural applicability of the results. Third, while the current study examined somatic symptoms as a moderator, other potentially important psychological and contextual moderators or mediators were not considered. Variables such as emotion regulation difficulties, trauma‐related cognitions and social support networks may critically shape the PTSD–pain relationship (Nickerson et al. 2015; Bonanno et al. 2011). Future research should adopt more comprehensive, multifactorial models to investigate these pathways. Furthermore, research has shown that individuals with high levels of somatic symptoms are more likely to experience severe and widespread pain. This suggests that somatic processes may amplify the link between PTSD and chronic pain by reinforcing maladaptive pain perceptions and increasing pain‐related distress (Morasco et al. 2013; Scarinci et al. 2003). While our findings emphasize the significance of somatic symptoms in this context, it is important to acknowledge that the study did not evaluate perceived stigma or attitudes towards mental health. As stigma is a significant factor in somatic symptoms, this omission is a limitation of the present study and must be addressed in future research.

## 7 Conclusion

This study provides important insights into the relationship between discrimination‐based chronic traumatic stress, PTSD, somatic symptoms and chronic pain among Syrian refugees residing in Türkiye. Our findings demonstrate that somatic symptoms significantly amplify the association between PTSD and chronic pain, highlighting the intricate biopsychosocial consequences of ongoing trauma exposure in forcibly displaced populations. By identifying both mediating and moderating pathways, this study emphasizes the need for integrative frameworks that account for the psychological, somatic and sociocultural dimensions of trauma‐related suffering. The results underline the importance of developing culturally responsive and trauma‐informed mental health interventions that extend beyond traditional PTSD treatments. While standard trauma‐focused approaches have shown effectiveness in many contexts, including refugee populations (Neuner et al. 2008; Turrini et al. 2019), our findings suggest that clinicians may benefit from considering the ongoing and identity‐based nature of discrimination‐related stressors. These factors may complicate symptom expression and clinical formulation and highlight the need for culturally responsive, trauma‐informed care.

Beyond clinical implications, the findings point to a broader public health imperative. Chronic pain and somatic distress can substantially impair functioning, limit workforce participation and hinder social integration. As such, health systems and humanitarian organizations must prioritize comprehensive care strategies that integrate physical, psychological and social support services. Enhancing healthcare accessibility, reducing mental health stigma and addressing structural barriers to care are essential steps towards improving long‐term outcomes for refugees globally.

## Ethics Statement

All procedures performed in this study involving human participants were in accordance with the ethical standards of the institutional and/or national research committee and with the 1964 Declaration of Helsinki and its later amendments or comparable ethical standards. Ethical approval was obtained from (MASKED FOR REVIEW), prior to data collection.

## Consent

Informed consent was obtained from all individual participants included in the study.

## Conflicts of Interest

The authors declare no conflicts of interest.

## Data Availability

The data that support the findings of this study are openly available in the Open Science Framework (OSF) at (MASKED FOR REVIEW).
